# Financial Outcomes Among Medicaid Expansion Enrollees

**DOI:** 10.1001/jamanetworkopen.2026.9328

**Published:** 2026-04-27

**Authors:** Nora V. Becker, Helen Levy, Richard A. Hirth, Sarah J. Clark, Renuka Tipirneni, John Z. Ayanian

**Affiliations:** 1 Division of General Medicine, Department of Internal Medicine, University of Michigan School of Medicine, Ann Arbor; 2 Institute for Healthcare Policy and Innovation, University of Michigan, Ann Arbor; 3 Institute for Social Research, University of Michigan, Ann Arbor; 4 Department of Health Management and Policy, University of Michigan School of Public Health, Ann Arbor; 5 Gerald R. Ford School of Public Policy, University of Michigan, Ann Arbor; 6 Department of Pediatrics, University of Michigan School of Medicine, Ann Arbor; 7 Child Health Research and Evaluation Center, University of Michigan School of Medicine, Ann Arbor

## Abstract

**Importance:**

Enrollment in Medicaid expansion may improve financial outcomes for low-income adults, but this association has not been studied in the longer-term postenrollment period.

**Objective:**

To estimate the association of enrollment in Medicaid expansion with financial outcomes for enrollees as long as 7 years after enrollment.

**Design, Setting, and Participants:**

This longitudinal cohort study used an event study analysis to compare changes in credit outcomes after relative to before enrollment, controlling for changes in outcomes over time, differences between individuals who enroll at different times, and linear trends in outcomes prior to enrollment. Participants included first-time enrollees in the Healthy Michigan Plan (HMP), Michigan’s Medicaid expansion plan, between April 1, 2014, and December 31, 2017. Enrollees were linked with their credit reports from a large national credit agency at 6-month intervals from 2013 to 2021, with outcomes obtained on the final business day of January and July of each calendar year. Data were analyzed from January 4, 2023, to December 9, 2025.

**Exposure:**

Enrollment in HMP.

**Main Outcomes and Measures:**

The event study analysis compared changes in 4 credit outcomes: medical debt in collections, nonmedical debt in collections, a subprime credit score (<600), and bankruptcy in the prior 2 years.

**Results:**

The study cohort included 575 283 enrollees (308 814 male [53.7%]; mean [SE] age, 42.1 [10.6] years). HMP enrollment was associated with large reductions in medical debt in collections (from −$101.9 [95% CI, −$127.6 to −$76.3] in postenrollment quarter 8 to −$983.0 [95% CI, −$1090.8 to −$875.1] in postenrollment quarter 29) and rates of subprime credit scores (from −0.038 [95% CI, −0.041 to −0.035] in postenrollment quarter 8 to −0.234 [95% CI, −0.247 to −0.221] in postenrollment quarter 29), with the magnitude of association increasing over time. No associations were observed between HMP enrollment and nonmedical debt in collections or bankruptcy.

**Conclusions and Relevance:**

In this cohort study of HMP enrollees, Medicaid expansion enrollment was associated with reductions in medical debt in collections and rates of subprime credit scores as long as 7 years after enrollment. These results suggest enrollment in Medicaid expansion was associated with excellent protection from out-of-pocket costs of medical care and overall improved financial stability, which in turn may be associated with improvements in enrollees’ health and well-being.

## Key Points

**Question:**

Is Medicaid expansion enrollment associated with subsequent financial outcomes for low-income adults?

**Findings:**

In this cohort study of 575 283 participants, Medicaid expansion enrollees in Michigan experienced significant reductions in medical debt in collections and rates of subprime credit score after enrollment.

**Meaning:**

These findings suggest that enrollment in Medicaid expansion may provide excellent protection from out-of-pocket costs of medical care and improved financial stability, which in turn may lead to improvements in enrollees’ health and financial well-being.

## Teaser

This cohort study estimates the association between Medicaid expansion enrollment and long-term financial outcomes among low-income adults who enrolled in the Healthy Michigan Plan.

## Introduction

The expansion of Medicaid under the Affordable Care Act (ACA) has enabled more than 21 million low-income adults to gain health insurance^1^ and has been associated with improved access to health care and reduced mortality for enrollees.^2,3^ It is crucial to examine the financial impact of enrollment in Medicaid because financial distress is associated with poor health outcomes across numerous patient populations.^4,5,6,7,8^

Enrollment in Medicaid can produce financial benefits for enrollees via 2 mechanisms. The first is an immediate reduction in the financial burden of the cost of medical care, and the second is longer-term improvements in health resulting in higher income. Research on earlier Medicaid expansions in the 1980s found that people enrolled in Medicaid in childhood had higher incomes, used fewer low-income tax credits, and paid more in taxes in adulthood.^9^

Several studies have examined the impact of immediate reductions in the costs of medical care after enrollment in Medicaid expansion plans. However, much of the work examining this question has used repeated cross-sections of survey data, which did not include the same individuals longitudinally, or data for which Medicaid enrollment status was not truly known—for instance, state or other area-level data—and thus included many people who were not enrolled in Medicaid.^10,11,12,13,14^ These studies have found that Medicaid expansion was associated with immediate improvements in patients’ financial circumstances. Two prior studies with individual-level enrollment data and longitudinal financial outcomes have shown that enrollment in Medicaid was associated with short-term improved credit outcomes for 21 months after enrollment,^15,16^ but to our knowledge, these outcomes have not been studied using longer-term data for individual Medicaid enrollees.

In April 2014, Michigan expanded Medicaid under the ACA, allowing adults with incomes at or below 138% of the federal poverty level to enroll in the state’s Medicaid expansion program, the Healthy Michigan Plan (HMP).^17,18^ The objective of this study was to estimate the association of enrollment in HMP with longer-term financial outcomes for HMP enrollees.

## Methods

This cohort study was conducted as a federally authorized evaluation of a public program, so the University of Michigan and the Michigan Department of Health and Human Services Institutional Review Boards deemed it exempt from review and the need for informed consent. We followed the Strengthening the Reporting of Observational Studies in Epidemiology (STROBE) reporting guideline.

### Data Source and Study Population

We identified all nonelderly adults who enrolled in HMP during the first 4 years of program implementation (April 1, 2014, to December 31, 2017). We included HMP enrollees aged 26 to 62 years as of January 2014 to exclude those potentially eligible during the study period for dependent coverage before 26 years of age or Medicare after 65 years of age and excluded individuals who died within 3 years of their first month of enrollment to ensure every individual could have at least 3 years of postenrollment data. We also excluded individuals who had other sources of Medicaid coverage in the half-year period that they first enrolled in HMP. Enrollees were then linked their credit reports from a large national credit agency at 6-month intervals from 2013 to 2021, with credit outcomes obtained on the final business day of January and July of each calendar year.^19,20^ Further details on the credit data linkage are provided in the eMethods in Supplement 1. Individuals who matched into at least 1 period of the credit data were included in the analysis in the periods for which they had data available.

### Outcomes and Covariates

We examined 4 credit outcomes of HMP enrollees: amount of medical debt in collections, amount of nonmedical debt in collections, a subprime credit score (<600), and bankruptcy in the past 2 years. Medical and nonmedical debt in collections outcomes were measured in dollars, and individuals without any reported medical or nonmedical debts sent to collections were coded as having a balance of 0. Having a subprime credit score or declaring bankruptcy were binary outcomes. A very small number of individuals matched into the credit data but were coded as not having sufficient data to calculate a credit score; these individuals were included in the analysis and coded as not having a subprime credit score. All outcomes were available in January and July of each year, and the data were constructed at the person-half-year level with as many as 18 observations per person during the 9-year study period.

### Statistical Analysis

Data were analyzed from January 4, 2023, to December 9, 2025. To examine changes in credit outcomes after enrollment in HMP, we performed a longitudinal event study analysis^21^ comparing changes in each outcome after enrollment. Longitudinal event study analyses are a type of quasi-experimental method used to estimate the association between a given treatment and outcome when random assignment to a treatment is not feasible; they generally use a source of randomness in treatment that is plausibly unrelated to the outcome and thus minimize the potential bias in the estimated association between treatment and outcomes.^22,23^ Our analysis leverages variation in the timing of enrollment to estimate changes in credit outcomes from before to after enrollment, controlling for changes in outcomes over time, differences between enrollees who enroll at different times, and linear trends in outcomes prior to enrollment. In accordance with how event study models are described in the economics literature, we use the terms *calendar time* to refer to dates in years or months and *event time* to describe time relative to the first date of enrollment in HMP. Our primary regression specification included the following variables: (1) a linear event quarters trend in quarters relative to HMP enrollment, (2) interaction terms between the linear trend in event quarters and year of enrollment fixed effects, (3) postenrollment event quarter fixed effects, (4) month of enrollment fixed effects, and (5) calendar year fixed effects.

The postenrollment event quarter coefficients were our coefficients of interest and can be interpreted as the change in outcomes experienced by patients relative to the counterfactual pre-enrollment linear trend. Put another way, the postenrollment event study coefficients represent the change in each outcome for each quarter after enrollment, relative to before enrollment, after adjusting for linear trends in the outcomes in the pre-enrollment period. Our specification allows pre-enrollment trends to vary by year of enrollment (the interaction terms between the event quarter linear trend and year of enrollment fixed effects). The analysis also controlled for overall changes in outcomes over time (year fixed effects) and differences in outcomes among individuals who enrolled at different times (month of enrollment fixed effects). We excluded the period immediately prior to enrollment in HMP as a baseline for all individuals. A full regression specification for our primary model, along with additional discussion of the reasons for our choice of this model as our primary specification, is available in the eMethods in Supplement 1.

The key assumption of our model was that no factor is correlated with both the timing of an individual’s enrollment and postenrollment credit outcomes. This assumption could be violated if, for instance, an individual enrolls in Medicaid as a result of a new health condition that subsequently forces them to stop working, reducing their income and thus potentially worsening their credit outcomes. We refer to this potential source of bias as enrollment timing endogeneity. To the extent that this type of bias occurred in our analysis, we would expect it to bias us toward finding a positive association between enrollment in HMP and poor credit outcomes, which would cause us to underestimate any financial benefits of enrollment in HMP.

We conducted several data validation checks of the credit data. First, we examined differences in our sample between individuals who matched into every period of the credit data vs those who did not match at all or those who matched into some but not all of the credit periods. Second, to ensure the credit outcomes in our cohort resembled those of non-Michigan residents, we compared overall trends over time in our outcomes between HMP enrollees and a random sample of individuals drawn from nonexpansion states. Finally, we replicated the results of a prior event study analysis of Michigan HMP enrollees’ credit outcomes that used credit data through the end of 2016.^16^

We also conducted several sensitivity analyses to examine the robustness of our results to alternative model specifications. First, we explored the validity of our choice to model pre-enrollment outcomes using a linear trend by estimating a fully flexible event study specification to visually and statistically examine pre-enrollment trends. Second, we explored the robustness of our results to several alternative model specifications, including (1) excluding the interaction terms between the pre-enrollment linear trend and year of enrollment fixed effects, (2) excluding individuals who died during the study period, and (3) including a limited set of individual-level covariates from 2014, available from the credit agency. Third, we explored the statistical feasibility of including half-year fixed effects rather than year fixed effects in our model. Finally, to examine potential heterogeneity in our results, we ran our analyses separately for 2014-2015 enrollees vs 2016-2017 enrollees. Additional details about these sensitivity analyses, including full model specifications, are described in the eMethods in Supplement 1. Our analyses were conducted using Stata-MP, version 19 (StataCorp LLC). All analyses used 2-sided *t* tests and a statistical significance threshold of α ≤ .05.

## Results

### Cohort Characteristics 

Among the 575 283 HMP enrollees included in our study cohort, 266 469 (46.3%) were female and 308 814 (53.7%) were male, the mean (SE) age was 42.1 (10.6) years, and 259 332 (45.1%) enrolled in 2014 (Table). The mean (SE) percentage of the federal poverty limit was 33.0% (39.4%), and individuals remained enrolled for a mean (SE) of 35.7 (24.2) months. Among HMP enrollees matched to any period of credit data, 45 509 (7.9%) were partially matched, that is, missing 1 or more periods of credit data, and these individuals were included in our analysis for the periods in which they had available credit data.

**Table.** Characteristics of HMP Enrollee Cohort

Characteristic	No. (%) of enrollees
Total	575 283 (100)
Sex	
Female	266 469 (46.3)
Male	308 814 (53.7)
Age at time of enrollment, mean (SE), y	42.1 (10.6)
Mean federal poverty limit, mean (SE), %	33.0 (39.4)
Months of enrollment in HMP, mean (SE)	35.7 (24.2)
Year of enrollment	
2014	259 332 (45.1)
2015	150 361 (26.1)
2016	92 664 (16.1)
2017	72 926 (12.7)
Missing ≥1 period of data	45 509 (7.9)

Abbreviation: HMP, Healthy Michigan Plan.

### Cohort Construction

**Figure 1.**
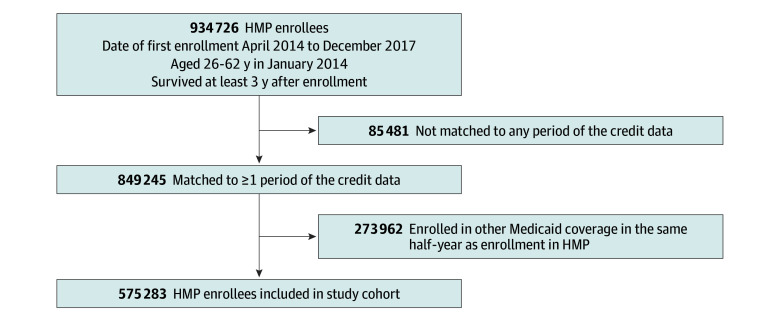
Study Cohort Flowchart HMP indicates Healthy Michigan Plan.

Individuals excluded from the analysis, and the reasons for exclusions, are shown in Figure 1. Observable characteristics of fully matched, partially matched, and unmatched individuals are presented in eTable 1 in Supplement 1. Of the HMP enrollees, 90.9% were linked to 1 or more periods of the credit data. After excluding individuals who were enrolled in other Michigan Medicaid coverage in the same half-year as they first enrolled in HMP, our final analytic cohort included 575 283 individuals.

### Unadjusted Changes in Credit Outcomes

We examined unadjusted outcomes among the HMP enrollees over time (eFigures 1-3 in Supplement 1) and relative to enrollment (eFigures 4-6 in Supplement 1). Notably, there was a large nonlinear increase in both medical and nonmedical debt in collections beginning in 2017 for all groups (eFigure 1 in Supplement 1), an increase that was particularly large for medical debt in collections.

### Data Validation Exercises

A comparison of fully matched, partially matched, and unmatched HMP enrollees is shown in eTable 1 in Supplement 1. Overall, individuals with partial or no credit data were more likely to be male and older, to have a lower income, and to have longer periods of enrollment in HMP. They also appeared to be in worse health, with more inpatient admissions, and individuals who were completely unmatched also had more chronic conditions compared with individuals with complete or partial credit data.

We confirmed that this nonlinear increase in debt in collections was not specific to HMP enrollees or Michigan residents and was present in the national data from this credit agency during this period (eFigures 7 and 8 in Supplement 1). We also replicated data from Miller et al^16^ with similar results; these results and additional details are available in eTable 2 in Supplement 1.

### Regression Analysis Results

**Figure 2.**
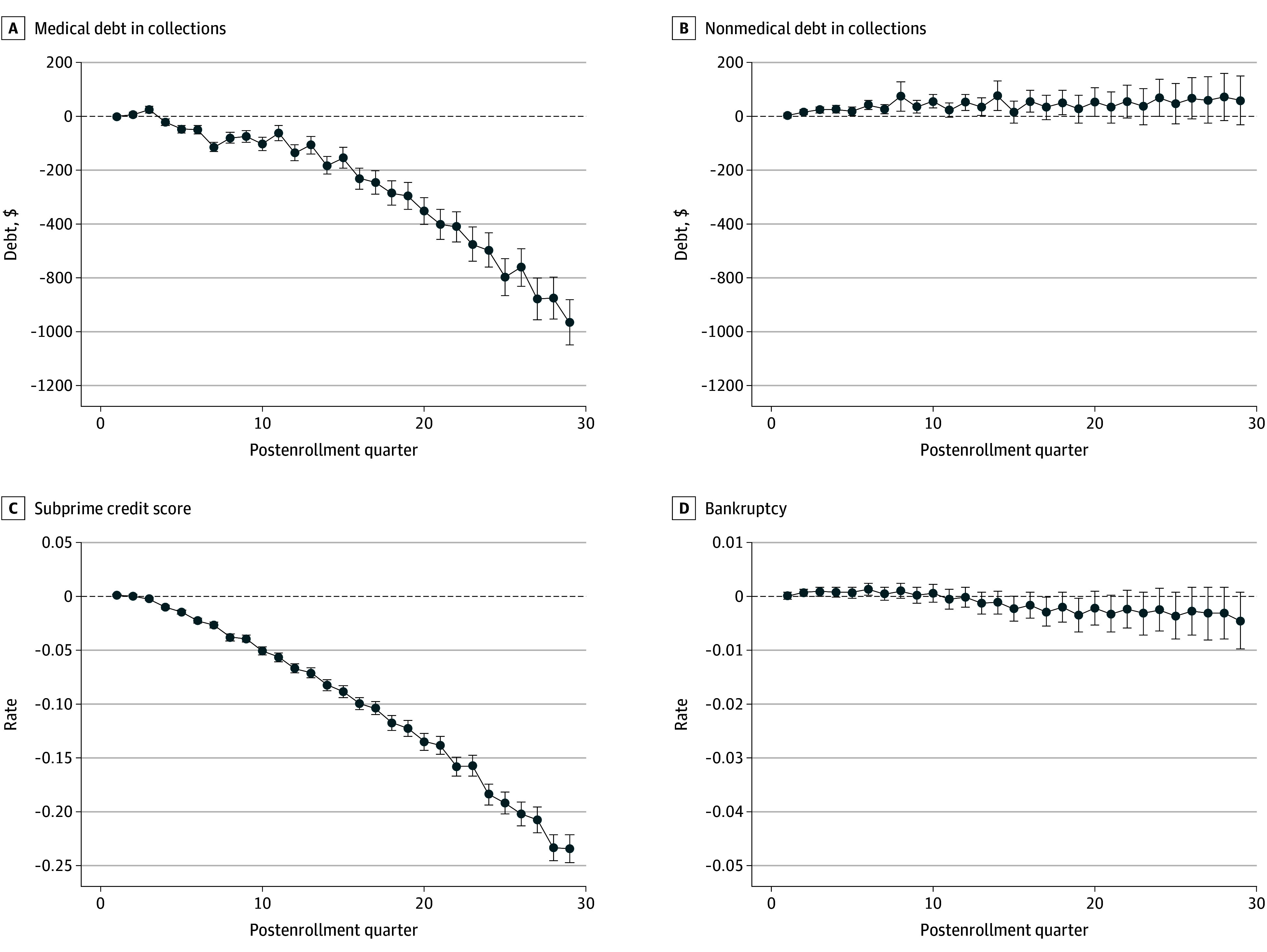
Event Study Coefficient Results From Primary Model Specification Line graphs display the regression coefficients (circles) and 95% CIs (error bars) for the set of postenrollment quarter fixed effects from our primary model specification. These results are presented numerically in eTable 3 in Supplement 1.

In our primary analysis, HMP enrollees experienced large and significant reductions in medical debt in collections after enrollment that grew over time, from −$101.9 (95% CI, −$127.6 to −$76.3; *P* < .001) in postenrollment quarter 8 to −$983.0 (95% CI, −$1090.8 to −$875.1; *P* < .001) in postenrollment quarter 29 (Figure 2A). In many postenrollment quarters, HMP enrollees did not experience significant changes in nonmedical debt in collections, although some postenrollment quarters showed small but statistically significant increases in nonmedical debt in collections in the range of $20 to $100 that did not persist over the study period (Figure 2B and eTable 3 in Supplement 1). HMP enrollees also experienced a large and significant reduction in rates of subprime credit score, from −0.038 (95% CI, −0.041 to −0.035; *P* < .001) in postenrollment quarter 8 to −0.234 (95% CI, −0.247 to −0.221; *P* < .001) in postenrollment quarter 29 (Figure 2C and eTable 3 in Supplement 1). In contrast, there were no consistent significant changes in rates of bankruptcy (Figure 2D). Regression coefficients from our primary model specification are displayed in eTable 3 in Supplement 1. For all outcomes, the linear event quarter trends were statistically significant, and for all outcomes except for bankruptcy, these pre-enrollment trends differed significantly by all enrollment years, demonstrating that pre-enrollment trends were both present and significantly different among enrollees who enrolled earlier vs later in the study period.

### Sensitivity Analyses

Our fully flexible event study model results are shown in eFigure 9 in Supplement 1 and demonstrate that all outcomes had a significant linear trend before enrollment, supporting our choice to include a linear trend in our primary model specification. In our exploration of including half-year fixed effects rather than year fixed effects in our model, we found that it was statistically infeasible to do so; additional details are available in the eMethods in Supplement 1.

Results for alternative model specifications are shown in eFigure 10 in Supplement 1. The results for medical debt in collections and subprime credit score were highly robust to alternative specifications of our primary model. For bankruptcy, HMP enrollees experienced small but significant decreases in bankruptcy in the specification where we omitted the interaction term between year of enrollment and the event quarter linear trend, on the order of 0.1 to 0.5 percentage points or 0.001 to 0.005 reductions in the rate of bankruptcy, but results for bankruptcy were mostly nonsignificant in all other specifications. Our results for nonmedical debt in collections were the most sensitive to model specification choice. In the specification where we omitted the interaction terms between the pre-enrollment linear trend and year of enrollment fixed effects, HMP enrollees demonstrated postenrollment reductions of several hundred dollars in nonmedical debt in collections. However, in subgroup analyses, the 2016-2017 enrollees experienced increases of several hundred dollars in nonmedical debt in collections relative to prior to enrollment (eFigure 11 in Supplement 1).

## Discussion

To our knowledge, this study is the first to examine longer-term financial outcomes for Medicaid expansion enrollees in a quasi-experimental framework as long as 7 years after enrollment; other studies have only examined outcomes up to 21 months after enrollment. These results are consistent with other studies of ACA Medicaid expansions using shorter-term data and either randomized or quasiexperimental research designs.^10,15,16,24^ Our results demonstrate that the early benefits in these outcomes seen in prior studies continued to accrue over time in our longer-term data. By 7 years after enrollment, the reductions in medical debt in collections and subprime credit score were very large and included a 30% to 50% relative reduction in rates of subprime credit score and, if we choose the peak average medical debt in collections seen over our study period, a 75% relative reduction in medical debt in collections.

Similar to pre-ACA Medicaid expansions, these results suggest that the ACA Medicaid expansions have been associated with large and long-term reductions in enrollees’ medical debt and large improvements in overall financial stability. Medical debt is associated with subsequent forgone medical care and worse self-reported physical and mental health^25,26^; reductions in medical debt may therefore contribute to corresponding improvements in individuals’ health outcomes.

We did not find any consistent change in bankruptcies after enrollment among HMP enrollees. Our estimates of the association of enrollment with nonmedical debt in collections were sensitive to our choice of cohort and model specification, making it difficult to draw confident conclusions about the association of HMP enrollment with nonmedical debt in collections.

### Limitations

This study has several limitations. First, we used observational data that may have been subject to potential confounding via enrollment timing endogeneity. To the extent that there may have been bias in our estimates, we expected that it would bias us toward associating enrollment in HMP with worsened financial circumstances. However, as our estimates on the association of enrollment with medical debt and rates of subprime credit score showed reductions in both outcomes, we can be confident in the direction of our estimates for these outcomes; this potential bias suggests that the magnitude of our estimates may be even greater than what our results suggest. Another potential source of selection bias comes from the exclusion of the small proportion of individuals who did not match into the credit data. These individuals appeared to be significantly different from individuals who partially or fully matched into the credit data, and thus this exclusion may limit the generalizability of our results, as they may not be applicable to individuals with no credit data over time.

Second, our analysis uses data from a single state, as national credit data linked to Medicaid expansion enrollees are unavailable. The regulatory requirements of the ACA’s Medicaid expansions meant that expansion plans were generally very similar, but not identical, across expansion states. While our results may therefore not be fully generalizable to other states’ Medicaid expansion programs, we would expect results from the Medicaid expansions in other states to be similar.

Third, there was a discontinuous increase in both medical and nonmedical debt in collections during our study period between 2016 and 2017 seen among all groups, potentially related to changes in reporting of debt in collections around this time. While we were able to validate that this increase was present nationally in the collections data from the credit agency, we were not able to ascertain the specific reason for this discontinuity, and it may be specific to the data from the credit agency from which we obtained our data.^27,28^ Our event study design controlled for this discontinuity by including year fixed effects but it may not have been able to fully adjust for it if this discontinuity varied significantly by month of enrollment. To the extent that this discontinuity may bias our results, we would expect it to bias us toward underestimating reductions in medical and nonmedical debt in collections associated with enrollment.

## Conclusions

In this cohort study using novel long-term individual-level credit data linked to Michigan Medicaid enrollees, enrollment in Medicaid expansion was associated with substantial reductions in medical debt and rates of subprime credit score. These reductions in medical debt may in turn be associated with improvements in enrollees’ ability to access care and their subsequent health and financial well-being. Future research should explore the mechanisms underlying these findings and longer-term changes in enrollees’ health outcomes that may result from improved financial stability after enrollment in Medicaid expansion.

## Notes

Supplement 1. **eMethods.** Credit Report Data, Regression Model Specifications, and Statistical Analyses
eReferences
**eTable 1.** Comparison of Matched, Unmatched, and Partially Matched HMP Enrollees **eFigure 1.** Unadjusted Debt Outcomes (Medical and Nonmedical Debt in Collections) Over Time Among HMP Enrollees **eFigure 2.** Rates of Subprime Credit Score Over Time Among HMP Enrollees **eFigure 3.** Rates of Bankruptcy Over Time Among HMP Enrollees **eFigure 4.** Unadjusted Debt Outcomes (Medical and Nonmedical Debt in Collections) Relative to Enrollment Among HMP Enrollees **eFigure 5.** Rates of Subprime Credit Score Relative to Enrollment Among HMP Enrollees **eFigure 6.** Rates of Bankruptcy Relative to Enrollment Among HMP Enrollees **eFigure 7.** Medical Debt in Collections Over Time for HMP Enrollees vs Comparison Sample **eFigure 8.** Nonmedical Debt in Collections Over Time for HMP Enrollees vs Comparison Sample **eTable 2.** Replication of Miller et al (2020) **eTable 3.** Regression Coefficients From Primary Analysis **eFigure 9.** Regression Results From “Fully Flexible” Event Study Specification **eFigure 10.** Regression Results From Alternative Model Specifications **eFigure 11.** Regression Results From 2014-2015 and 2016-2017 Enrollee Subgroups

Supplement 2.
Data Sharing Statement
