# Supplementary material for: Financial Outcomes Among Medicaid Expansion Enrollees
Source: JAMA Netw Open. 2026 Apr 27;9(4):e269328. doi: 10.1001/jamanetworkopen.2026.9328 (PMC13122400; doi:10.1001/jamanetworkopen.2026.9328)
Supplement: Supplement 1. — eMethods. Credit Report Data, Regression Model Specifications, and Statistical Analyses eReferences eTable 1. Comparison of Matched, Unmatched, and Partially Matched HMP Enrollees eFigure 1. Unadjusted Debt Outcomes (Medical and Nonmedical Debt in Collections) Over Time Among HMP Enrollees eFigure 2. Rates of Subprime Credit Score Over Time Among HMP Enrollees eFigure 3. Rates of Bankruptcy Over Time Among HMP Enrollees eFigure 4. Unadjusted Debt Outcomes (Medical and Nonmedical Debt in Collections) Relative to Enrollment Among HMP Enrollees eFigure 5. Rates of Subprime Credit Score Relative to Enrollment Among HMP Enrollees eFigure 6. Rates of Bankruptcy Relative to Enrollment Among HMP Enrollees eFigure 7. Medical Debt in Collections Over Time for HMP Enrollees vs Comparison Sample eFigure 8. Nonmedical Debt in Collections Over Time for HMP Enrollees vs Comparison Sample eTable 2. Replication of Miller et al (2020) eTable 3. Regression Coefficients From Primary Analysis eFigure 9. Regression Results From “Fully Flexible” Event Study Specification eFigure 10. Regression Results From Alternative Model Specifications eFigure 11. Regression Results From 2014-2015 and 2016-2017 Enrollee Subgroups [file jamanetwopen-e269328-s001.pdf]

# Supplementary Online Content

Becker NV, Levy H, Hirth RA, Clark SJ, Tipirneni R, Ayanian JZ. Financial outcomes among Medicaid expansion enrollees. *JAMA Netw Open.* 2026;9(4):e269328. doi:10.1001/jamanetworkopen.2026.9328

**eMethods.** Credit Report Data, Regression Model Specifications, and Statistical Analyses
**eReferences**
**eTable 1.** Comparison of Matched, Unmatched, and Partially Matched HMP Enrollees
**eFigure 1.** Unadjusted Debt Outcomes (Medical and Nonmedical Debt in Collections) Over Time Among HMP Enrollees
**eFigure 2.** Rates of Subprime Credit Score Over Time Among HMP Enrollees
**eFigure 3.** Rates of Bankruptcy Over Time Among HMP Enrollees
**eFigure 4.** Unadjusted Debt Outcomes (Medical and Nonmedical Debt in Collections) Relative to Enrollment Among HMP Enrollees
**eFigure 5.** Rates of Subprime Credit Score Relative to Enrollment Among HMP Enrollees
**eFigure 6.** Rates of Bankruptcy Relative to Enrollment Among HMP Enrollees
**eFigure 7.** Medical Debt in Collections Over Time for HMP Enrollees vs Comparison Sample
**eFigure 8.** Nonmedical Debt in Collections Over Time for HMP Enrollees vs Comparison Sample
**eTable 2.** Replication of Miller et al (2020)
**eTable 3.** Regression Coefficients From Primary Analysis
**eFigure 9.** Regression Results From “Fully Flexible” Event Study Specification
**eFigure 10.** Regression Results From Alternative Model Specifications
**eFigure 11.** Regression Results From 2014-2015 and 2016-2017 Enrollee Subgroups

This supplementary material has been provided by the authors to give readers additional information about their work.

© 2026 Becker NV et al. *JAMA Network Open*.

**eMethods.** Credit Report Data, Regression Model Specifications, and Statistical Analyses

1. *Linkage to credit report data*

We extracted data from the Michigan Department of Health and Human Services (MDHHS)'s data warehouse to identify all enrollees who enrolled in the Healthy Michigan Plan (HMP) at any point between April 2014 and December 2017, who were ages 26-62 in January 2014, and survived at least 3 years post-enrollment. These individuals were then linked to their credit reports at one of the three major U.S. credit agencies using name, most recent address, and social security number (SSN). All direct identifiers (name, address, and SSN) were removed from the dataset by the credit agency prior to delivery of the data back to the HMP evaluation team, and all subsequent analyses were done using a de-identified study ID created specifically for the purpose of the data linkage. The study team's crosswalk between the study ID and patient direct identifiers was permanently deleted per the data use agreement with the credit agency.

To mask the identity of HMP enrollees, the file sent to the credit agency also included 200,000 individuals randomly selected from the Michigan Care Improvement Registry (MCIR), another state health database. The MCIR database includes any Michigan residents who have received a vaccine, and contains names and addresses; SSNs were randomly generated for these individuals as well. SSNs for the MCIR population were generated as follows: an SSN has three components, AAA-GG-SSSS. AAA is the area code, GG is the group code, and SSSS is the serial number. The generated SSNs were created by sampling randomly from the area codes in the Healthy Michigan Plan data and combining each area code with a randomly generated group code where the sampling is capped to be less than the maximum even-numbered group code observed in the Healthy Michigan Plan data in the associated area. The last four digits (SSSS) were generated at random. Constructing randomly generated social security numbers in this way assured that the generated numbers correspond to real individuals. The order of the MCIR names within the dataset were then randomized to ensure that the identities of HMP enrollees were masked and not identifiable by the credit agency. Because these SSNs did not match their corresponding names and addresses, the individuals drawn from the MCIR database did not successfully match into the credit agency's database and were not included in any subsequent analyses.

We also pulled a comparison sample for HMP enrollees from the credit agency's national data. One million individuals living in the 13 states that had not expanded Medicaid as of 2021 (AL, FL, GA, KS, MO, MS, NC, SC, SD, TN, TX, WI, and WY) were selected randomly from the credit agency's national database, and credit data for the same periods were obtained for these individuals. To identify a population of comparison individuals analogous to HMP enrollees, this sample was restricted to individuals ages 26–62 in January 2014 with an estimated household income of <$35,000 per year, the approximate HMP income eligibility threshold for a family of four during our study period.[1] Estimated household income was derived from a proprietary algorithm available from the credit agency, and calculated using a proprietary imputation algorithm validated against income tax filings for a subset of its national database. All three inclusion criteria (state of residence, age, and estimated income) for the comparison sample were obtained in the data period January 2014.

Note: This comparison sample was originally intended to be used as a control group in a planned difference-in-differences (DiD) analysis, however, attempted DiD analyses showed consistent violations of the parallel trends assumption and thus we ultimately converted our analysis to the current event study approach without a control group.

2. *Primary and alternative regression model specifications*

Our primary event study model specification is shown in Equation 1:

$$Y_i = \partial(e * \gamma_c) + \beta_e(e \geq 1) + \alpha_c + \theta_y + \epsilon_{itc} \quad (1)$$

Here, $i$ represents individuals, $\alpha_c$ represents month of enrollment fixed effects (FE), and $\theta_y$ represents calendar year FE. The primary coefficients of interest are a linear trend in event time ($e$) interacted with a set of year of enrollment FE ($\gamma_c$), and then a set of post-enrollment event quarter fixed effects for post-enrollment quarters 1-

© 2026 Becker NV et al. *JAMA Network Open*.

29 ($\beta_e$). Note that because our credit outcomes are measured at the half-year level, but our event time is at the quarter level, half of our cohort contributes to the coefficients for odd-numbered event quarters (-5, -3, -1, 1, 3, 5 etc.), while the other half contributes to the coefficients for even-numbered event quarters (-4, -2, 0, 2, 4, etc.). We exclude the quarter immediately prior to enrollment for all individuals; practically speaking this means we exclude event quarter -1 for the half of the cohort contributing to the odd-numbered event study coefficients, and event quarter 0 for the half of the cohort contributing to the even-numbered event study coefficients. We estimated all models using ordinary least squares and reported heteroskedasticity-robust standard errors clustered at the individual level. Results from this primary model are reported graphically in Figure 2 in the manuscript and eTable 3 in this Supplement.

Fundamentally, our analysis faced two methodological challenges not encountered by the Miller et. al.[2] study we replicate below. First, there is the issue of the discontinuous jump in medical and non-medical debt in collections between 2016 and 2017. We discuss this in greater detail below, but it may be the result of a change in how collections data were reported by the credit agency at that time. We have dealt with this discontinuity by adding time fixed effects to our models. Second, there is the issue of potential endogeneity in enrollment timing, particularly among later years of enrollees, who may be enrolling because they have experienced a new health or financial shock that makes them newly eligible for HMP and that may also be correlated with their post-enrollment outcomes. We have controlled for this by allowing pre-enrollment linear trends to vary by year of enrollment; to the extent that this does not fully control for endogeneity of enrollment timing, we believe this biases us towards the null for our key findings.

We explored the validity of our decision to model pre-enrollment outcomes using a linear trend by estimating a "fully flexible" event study specification as shown in Equation 2:

$$Y_i = \beta_e + \alpha_c + \theta_y + \epsilon_{itc} \qquad 2$$

Again, here $i$ represents individuals, $\alpha_c$ represents month of enrollment fixed effects, and $\theta_y$ represents calendar year fixed effects. $\beta_e$ are now a full set of pre- and post-enrollment event time fixed effects, and each event study coefficient now represents the change in each outcome relative to the reference period, which is again the observation immediately prior to enrollment for each enrollee. The full set of pre- and post-enrollment event study coefficients from Equation 2 are shown graphically in eFigure 9, and show that for each outcome, there is a clear pre-enrollment linear trend, supporting our decision to include linear trends in our primary specification.

To test the robustness of our primary specification results, we ran our analysis using several alternative specifications or subgroups. First, we ran our analysis using Equation 1, but excluding individuals who died at any point during the study period beyond the initial three-year survival we required as an inclusion criteria. Both the MDHHS data and the credit data included a flag for deceased status in each half-year period; in this sensitivity analysis we excluded anyone who had one or both of those flags at any point in our study period.

Second, we ran our analysis on the full cohort using Equation 1, with the addition of a limited set of time-invariant covariates available from the credit agency as of January 2014, all of which were included as categorical fixed effects: Sex (female vs male), marital status (married, single, or missing), education level (less than high school, high school diploma, some college, bachelor's degree, graduate degree, or missing), homeowner (yes vs missing), and renter (yes vs missing). The variables homeowner and renter had "no" and "missing" categories grouped together in the raw data. With the exception of sex, which was taken from MDHHS data and was therefore complete for all HMP enrollees, many of these variables were missing for a significant subset of HMP enrollees, and so missing was included as a category. However, as all of these covariates were taken from January 2014, this sensitivity analysis did exclude a small number of HMP enrollees who did not match into the January 2014 credit data.

Results from these two sensitivity analyses above were almost identical to the results from our primary specification and are shown graphically in eFigure 10.

© 2026 Becker NV et al. *JAMA Network Open*.

Third, we ran a sensitivity analysis where we included only a single linear event quarter trend and removed the interaction terms between the linear event quarter trend and year of enrollment, as described in Equation 3:

$$Y_i = \partial e + \beta_e(e \geq 1) + \alpha_c + \theta_y + \epsilon_{itc} \qquad 3$$

Results from this analysis are also presented graphically in eFigure 10. Results from this analysis were very similar to our primary specification for medical debt in collections and subprime credit score, although the estimates were somewhat smaller in magnitude. Results for this specification did differ from our primary specification for bankruptcy, where we saw similar magnitude estimates of small reductions in bankruptcy rates as in our primary models, but in this specification these reductions were statistically significant. And for non-medical debt in collections, this specification showed significant reductions in non-medical debt in collections of several hundred dollars.

Finally, to examine potential heterogeneity by year of enrollment, we ran Equation 1 separately for the 2014-2015 enrollees and the 2016-2017 enrollees. Those results are shown graphically in eFigure 11. Results for both subgroups are very similar for all outcomes except for non-medical debt in collections, where the 2014-2015 subgroup saw no significant changes, but the 2016-2017 subgroup saw increases in non-medical debt post-enrollment of several hundred dollars.

Overall, our interpretation of these sensitivity analyses is that our results are extremely robust for the outcomes of medical debt in collections, subprime credit score, and bankruptcy, but that our results for non-medical debt in collections are sensitive to choice of subgroup and model specification and cannot be interpreted with as much confidence.

3. *Replication of Miller et. al. (2020)*[3]

As a data validation exercise, we replicated the results of Miller et. al. (2020), an earlier study of credit outcomes among HMP enrollees that used a similar event study analysis to examine changes in credit outcomes through the end of 2016. That study, like ours, linked HMP enrollees to half-year credit data observations in January and July, using data from 2011-2016. Of note, Miller et. al. used data from a different credit agency than the one that provided our data.

To most closely replicate Miller et. al., we limited our replication cohort to individuals who enrolled between April 2014 and December 2015, the same population used in that study. That study also used slightly different primary outcomes than we did in our study. Like our study, they examined medical debt in collections and rates of subprime credit score, but they used total debt in collections (rather than non-medical debt in collections) and a count of total bankruptcies on an individual's credit file (rather than a binary indicator for an individual having filed for bankruptcy in the past two years). These two alternative outcomes, while not our primary outcomes, were also available in our data, so we used them in this replication exercise.

Miller et. al. also used a different event study specification, shown in Equation 4:

$$Y_i = \beta_e + \alpha_c + \varphi_m + \epsilon_{itc} \qquad 4$$

This specification is very similar to our fully flexible event study specification in Equation 2, with month of enrollment fixed effects ($\alpha_c$), and $\beta_e$ again representing a full set of pre- and post-enrollment event time fixed effects. However, Miller et. al. did not include the calendar year FE that we include in Equation 2 ($\theta_y$), instead including a single dummy variable for whether or not an observation took place in January or July ($\varphi_m$). While the authors of that study don't mention whether they explored including a set of calendar time FE, given the discontinuity in the debt in collections outcomes we observed in our data, it was crucial that our models adjusted for this discontinuity by including year FE.

© 2026 Becker NV et al. *JAMA Network Open*.

We replicated Equation 4 using our data. Of note, Miller et. al. also ran their analysis adjusting for an event quarter linear trend, as we do in Equation 3 above. However, their analysis included data back through 2011, while our data only goes back through 2013, and thus we would not expect any modeled pre-enrollment linear trends to necessarily match. Equation 4 from Miller et. al., which allows for a fully flexible specification and therefore only compares each post-enrollment event study coefficient to the baseline period immediately prior to enrollment, was therefore the closest replication we could perform.

Miller et. al. reported results from Equation 4 for post-enrollment quarters 2, 4, and 7, as seven post-enrollment quarters were the last included in their data. They do not report the pre-enrollment event study coefficients from this model specification or include any figures that show these coefficients. We display our full set of event study coefficients for Equation 4 alongside the coefficients from post-enrollment quarters 2, 4, and 7 reported by Miller et. al. in eTable 2. For reference, these are the coefficients reported in the first columns for each outcome in Tables 3 and 4 in their manuscript. Overall they are similar; the directions and statistical significance match for all coefficients, although the magnitudes of our estimates are somewhat smaller than those reported by Miller et. al. This may be a result of differences in data collection and reporting across credit agencies, as they obtained their data from a different credit agency than we did for our study. There may also have been retrospective modifications to how these historical data are reported, since Miller et. al. linked their data in 2017, and our linkage was done in 2022. Regardless, we find this similarity to the results from Miller et. al. very reassuring.

It is also important to note that for the outcomes of medical and non-medical debt in collections in our estimates in eTable 2, the magnitude of the event study coefficients reverse dramatically for post-enrollment quarter 8, going from strongly negative to strongly positive between post-enrollment quarters 7 and 8. Post-enrollment quarter 8 is the first post-enrollment quarter than incorporates data from January 2017, the period in which the discontinuity occurs in our data, and thus underscores the importance of using a primary specification that includes calendar time fixed effects, as we do in all of our models.

4. *Statistical feasibility of including half-year fixed effects in our model*

Given the large discontinuity in our debt outcomes between 2016 and 2017 and the importance of including some type of calendar time FE in our model, we explored the feasibility of including half-year FE in our primary model. Including half-year FE in our event study models would require an additional normalization, as the inclusion of a full set of event, cohort, and time FEs in event study models introduces a perfect collinearity. This can be intuited by the fact that, if one is given any two of these values for an individual, one can calculate the third; for instance, if we know that someone enrolled in HMP in April 2014, and we are told that the time period of a specific observation is January 2015, we can calculate that this observation is event quarter 3 for that individual. There is an excellent discussion of this in a recent methodologic introduction to event study models,[2] and several event study models in the health economics literature have used and discussed this type of additional normalization.[4,5] A common additional normalization used in these studies is to group several pre-event time periods into a single pre-event period. The general consensus in the literature is that, when using one of these additional normalizations, that one should ensure that the results are robust to the choice of normalization, as there is no a priori best option and the choice of normalization is untestable.[2]

Unfortunately, we found that our event study coefficient estimates from specifications including half-year FE were both highly sensitive to the choice of normalization, and very imprecise with extremely wide confidence intervals, raising the concern for significant multicollinearity in our model. An examination of the variance inflation factors (VIFs)[6] for versions of the model including half-year FE supported this, with VIFs in the 1000s for many of the FE coefficients. While VIF cut-offs are debatable, VIFs in the 1000s suggests extreme levels of multicollinearity, and were also several orders of magnitude higher than our primary model specification with

© 2026 Becker NV et al. *JAMA Network Open*.

only year FE. We therefore concluded that a model including half-year FEs was statistically infeasible and used year FEs in both our primary and alternative model specifications.

5. *Discontinuous increase in medical and non-medical debt in collections in 2017*

A review of the temporal trends in medical and non-medical debt outcomes in our data showed a significant discontinuous increase in both outcomes beginning in January 2017 (eFigure 1). As this discontinuous increase does not appear to be present in other national credit data sources,[7,8] we used the comparison sample from non-Michigan states to validate that this increase was not specific to Michigan or HMP enrollees. We found that the comparison sample experienced very similar increases in 2017 to those experienced by HMP enrollees (eFigures 7 and 8), confirming that this increase occurred nationally in the data and was not specific to our study population. We were not able to ascertain the reason for this discontinuity, and it may be specific to the data from the credit agency from which we obtained our data. Our event study design controls for this discontinuity by including year fixed effects. To the extent that this discontinuity may bias our results, we would expect it to bias us towards understimating reductions in medical and non-medical debt in collections associated with enrollment.

**eReferences**


1. Prior HHS Poverty Guidelines and Federal Register References. Office of the Assistant Secretary for Planning and Evaluation (ASPE), Department of Health and Human Services. Accessed March 21, 2025. http://aspe.hhs.gov/topics/poverty-economic-mobility/poverty-guidelines/prior-hhs-poverty-guidelines-federal-register-references

2. Miller DL. An Introductory Guide to Event Study Models. *Journal of Economic Perspectives*. 2023;37(2):203-230. doi:10.1257/jep.37.2.203

3. Miller S, Hu L, Kaestner R, Mazumder B, Wong A. The ACA Medicaid Expansion in Michigan and Financial Health. *Journal of Policy Analysis and Management*. 2020;40(2). doi:https://doi.org/10.1002/pam.22266

4. Dobkin C, Finkelstein A, Kluender R, Notowidigdo MJ. The Economic Consequences of Hospital Admissions. *American Economic Review*. 2018;108(2):308-352. doi:10.1257/aer.20161038

5. Gross T, Notowidigdo MJ, Wang J. The Marginal Propensity to Consume over the Business Cycle. *American Economic Journal: Macroeconomics*. 2020;12(2):351-384. doi:10.1257/mac.20160287

6. Thompson CG, Kim RS, Aloe AM, Becker BJ. Extracting the Variance Inflation Factor and Other Multicollinearity Diagnostics from Typical Regression Results. *Basic and Applied Social Psychology*. 2017;39(2):81-90. doi:10.1080/01973533.2016.1277529

7. Kluender R, Mahoney N, Wong F, Yin W. Medical Debt in the US, 2009-2020. *JAMA*. 2021;326(3):250-256. doi:10.1001/jama.2021.8694

8. The Changing Medical Debt Landscape in the United States. Urban Institute. Accessed December 4, 2025. https://apps.urban.org/features/medical-debt-over-time/


© 2026 Becker NV et al. *JAMA Network Open*.

**eTable 1.** Comparison of Matched, Unmatched, and Partially Matched HMP Enrollees

| | Matched to all periods | Matched to 1-17 periods | Unmatched |
|---|---|---|---|
| Total (N, %) | 779,180 | 70,065 | 85,481 |
| Number of missing periods, N (%) | | | |
| 0 | 779,180 (100) | | 0 |
| 1-2 | 0 | 23,502 (33.5) | 0 |
| 3-6 | 0 | 24,774 (35.4) | 0 |
| 7-17 | 0 | 21,789 (31.1) | 0 |
| 18 | 0 | | 85,481 (100) |
| Sex, N(%) | | | |
| Female | 418,240 (53.7) | 28,133 (40.2) | 34,744 (40.7) |
| Male | 360,940 (46.3) | 41,932 (59.9) | 50,737 (59.4) |
| Age at time of enrollment, mean (SE) | 41 (10.2) | 41 (10.6) | 45 (10.2) |
| Average % federal poverty limit, mean (SE) | 33 (36.9) | 28 (35.3) | 20 (32.5) |
| Months of enrollment in HMP, mean SE) | 34 (24.4) | 37 (25.0) | 42 (27.7) |
| Average ED visits/year | 3.5 (24.9) | 3.2 (27.4) | 3.3 (23.8) |
| Average inpatient admissions/year | .5 (4.2) | .6 (4.7) | .8 (5.3) |
| Average number of body systems with chronic conditions | 4.9 (3.7) | 4.8 (3.7) | 5.5 (3.9) |
| Year of enrollment, N(%) | | | |
| 2014 | 374,509 (48.1) | 31,477 (44.9) | 45,358 (53.1) |
| 2015 | 183,876 (23.6) | 16,120 (23.0) | 18,443 (21.6) |
| 2016 | 124,625 (16.0) | 12,177 (17.4) | 12,186 (14.3) |
| 2017 | 96,170 (12.3) | 10,291 (14.7) | 9,494 (11.1) |

© 2026 Becker NV et al. *JAMA Network Open*.

**eFigure 1.** Unadjusted Debt Outcomes (Medical and Nonmedical Debt in Collections) Over Time Among HMP Enrollees

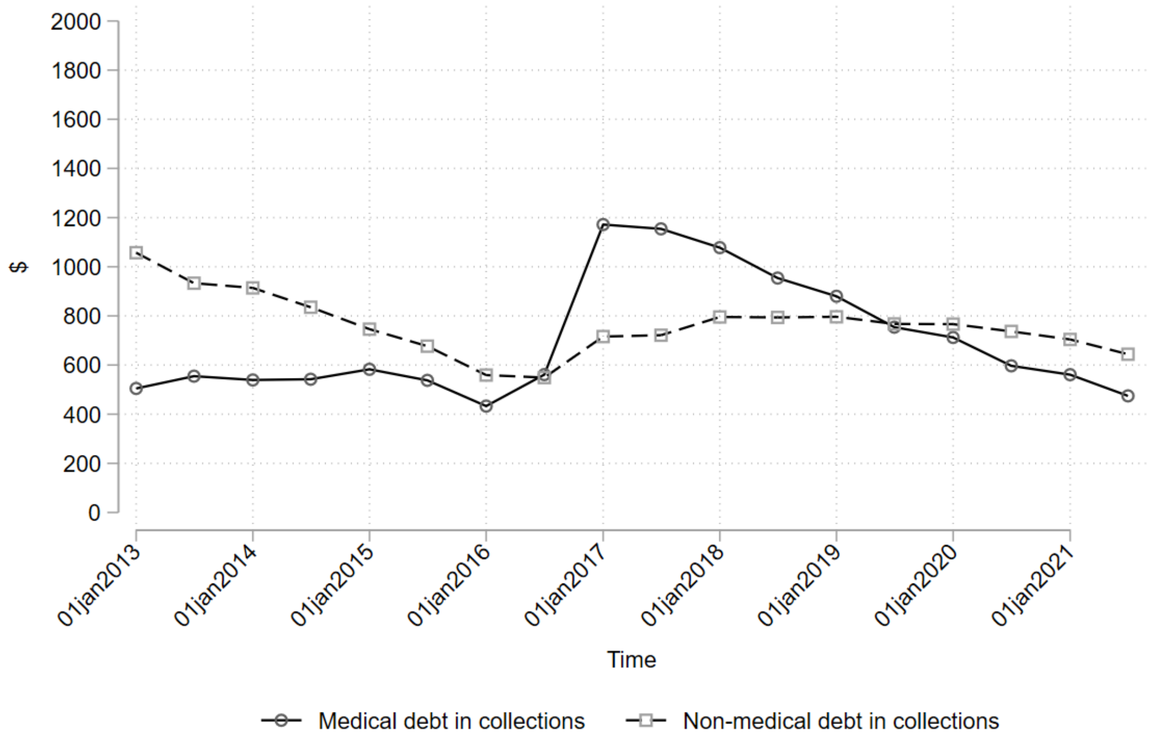


**eFigure 2.** Rates of Subprime Credit Score Over Time Among HMP Enrollees

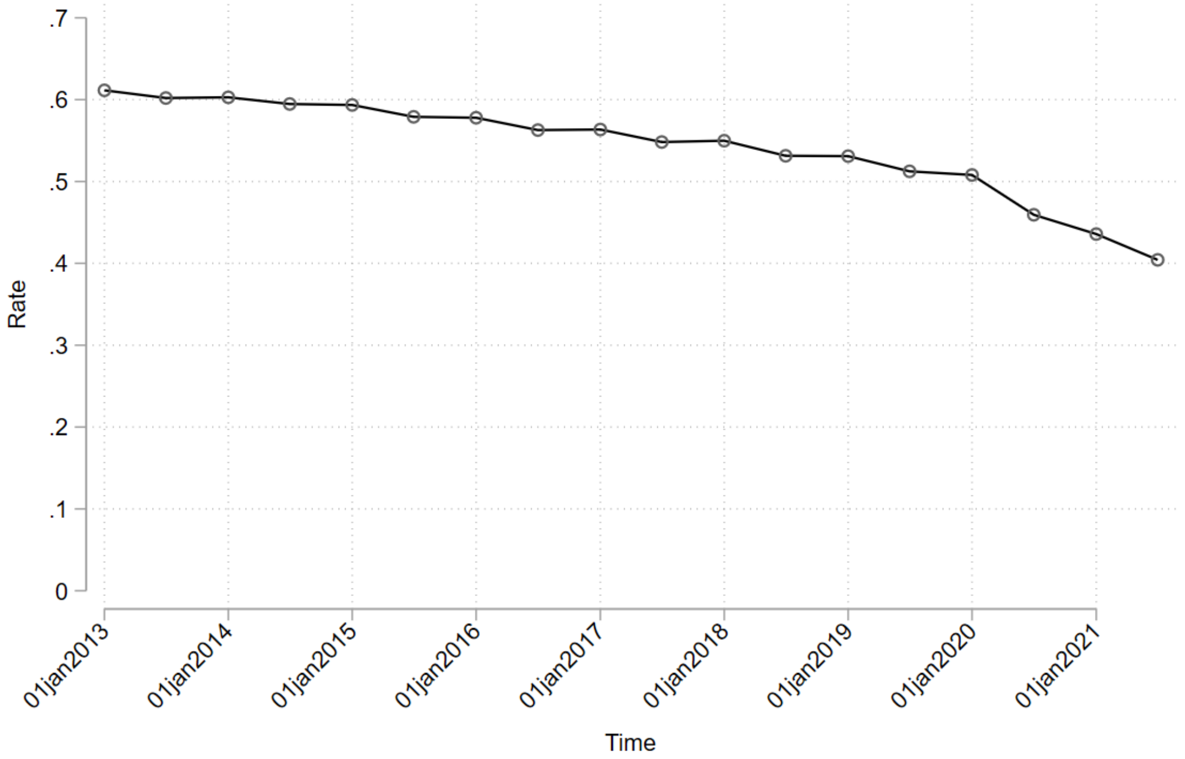


© 2026 Becker NV et al. *JAMA Network Open*.

**eFigure 3.** Rates of Bankruptcy Over Time Among HMP Enrollees

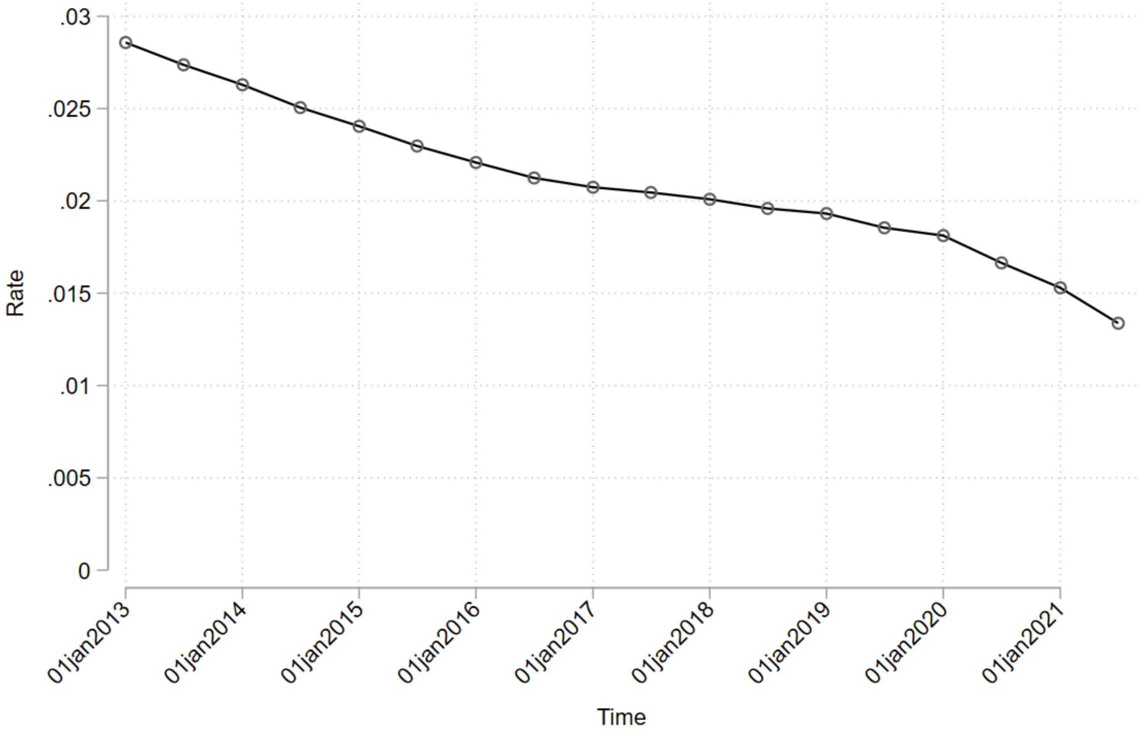


© 2026 Becker NV et al. *JAMA Network Open*.

**eFigure 4.** Unadjusted Debt Outcomes (Medical and Nonmedical Debt in Collections) Relative to Enrollment Among HMP Enrollees

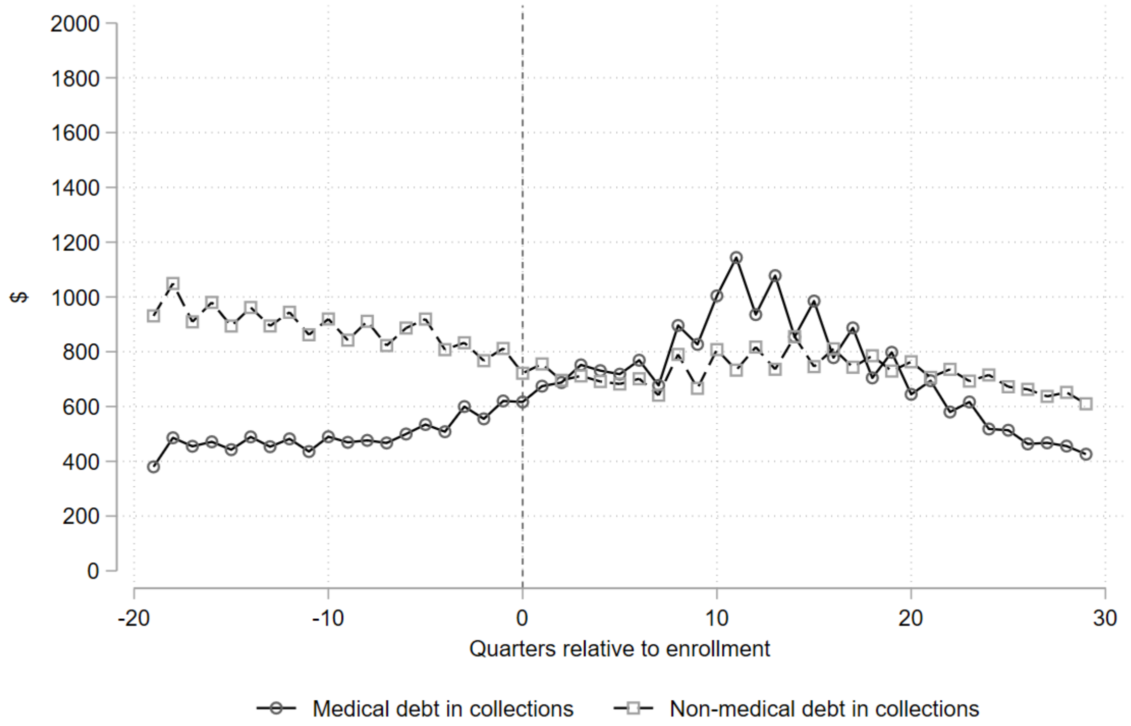


**eFigure 5.** Rates of Subprime Credit Score Relative to Enrollment Among HMP Enrollees

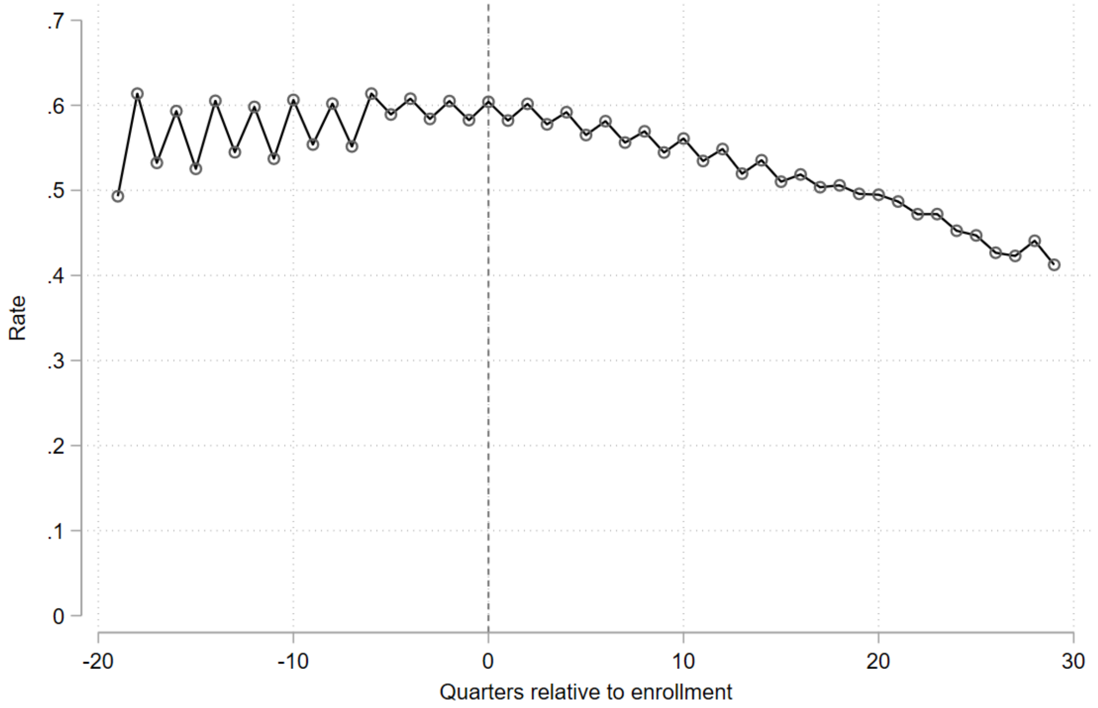


© 2026 Becker NV et al. *JAMA Network Open*.

**eFigure 6.** Rates of Bankruptcy Relative to Enrollment Among HMP Enrollees

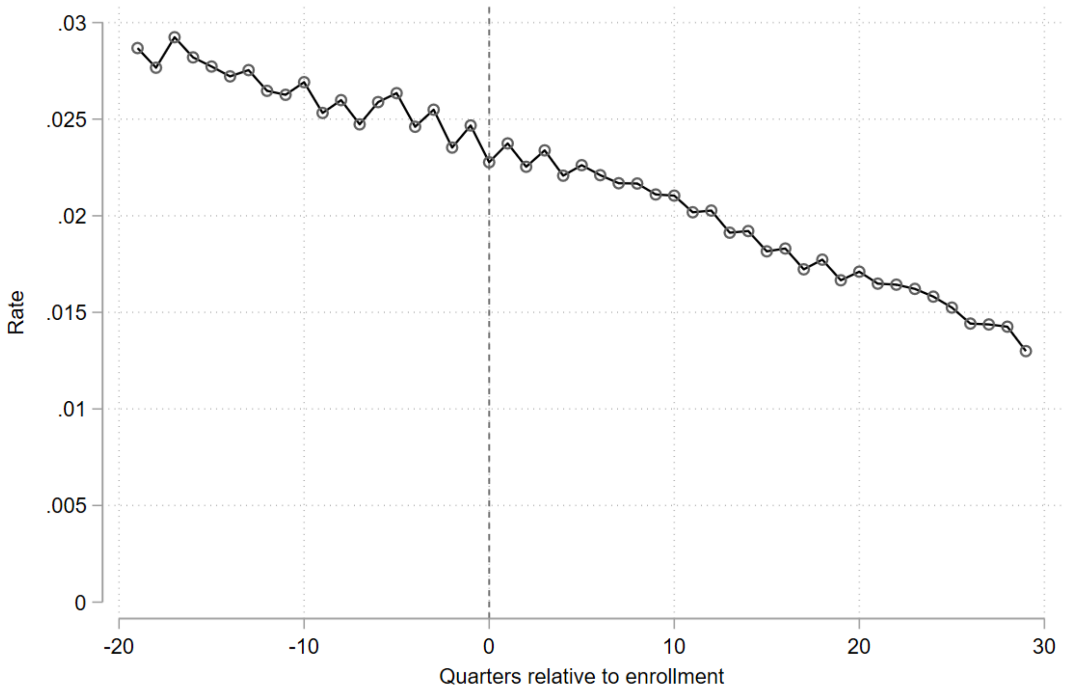


© 2026 Becker NV et al. *JAMA Network Open*.

**eFigure 7.** Medical Debt in Collections Over Time for HMP Enrollees vs Comparison Sample

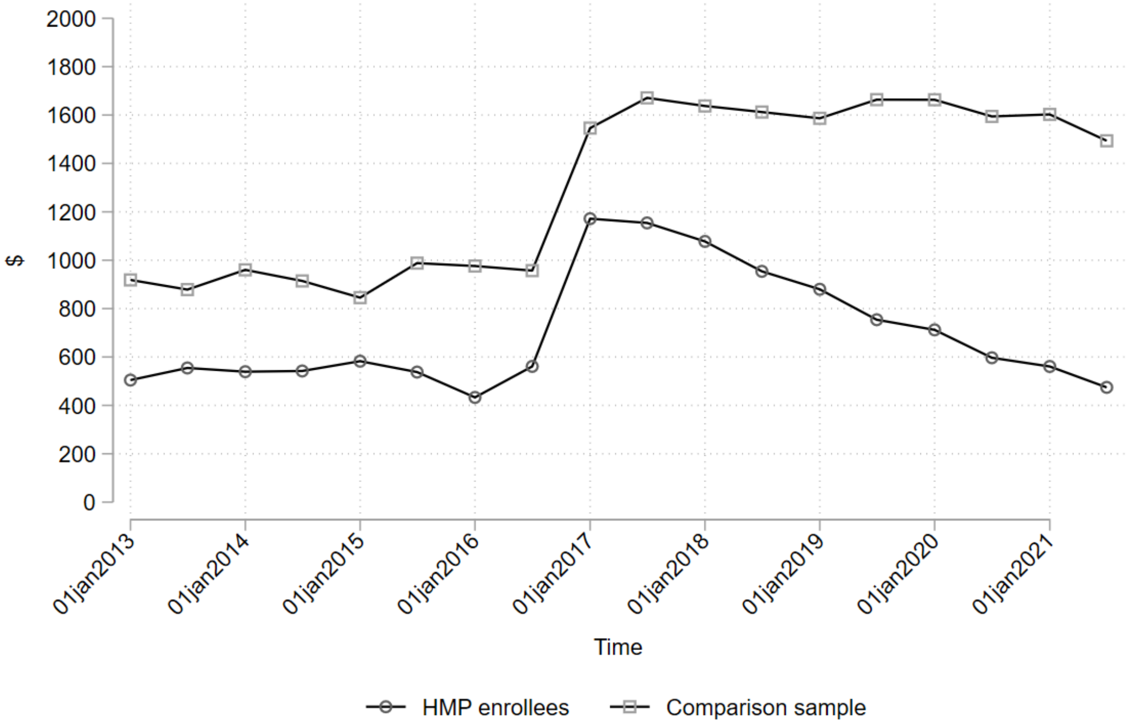


**eFigure 8.** Nonmedical Debt in Collections Over Time for HMP Enrollees vs Comparison Sample

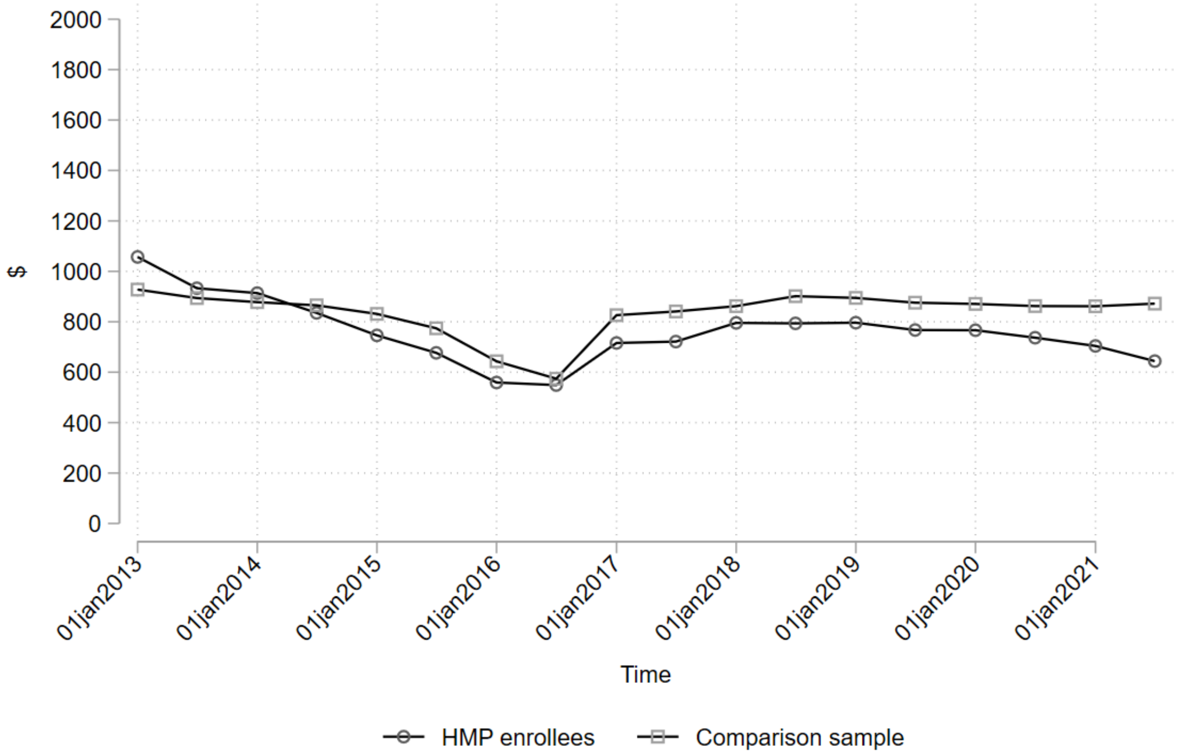


© 2026 Becker NV et al. *JAMA Network Open*.

**eTable 2.** Replication of Miller et al (2020)

| | Medical debt in collections | | Total balance on collections | | Total number of public record bankruptcies | | Subprime credit score | |
|---|---|---|---|---|---|---|---|---|
| | Replication result | Miller result | Replication result | Miller result | Replication result | Miller result | Replication result | Miller result |
| Pre-enrollment quarter -8 | -48.7 (-72.9 - - 24.6) | | 222.6 (183.2 - 261.9) | | 0.0062 (0.0042 - 0.0082) | | 0.016 (0.013 - 0.019) | |
| Pre-enrollment quarter -7 | -18.5 (-42.4 - 5.49) | | 90.6 (51.5 - 129.6) | | 0.0023 (-0.0000049 - 0.0045) | | 0.0076 (0.0046 - 0.011) | |
| Pre-enrollment quarter -6 | -18.4 (-40.2 - 3.36) | | 197.2 (167.7 - 226.7) | | 0.0043 (0.0031 - 0.0055) | | 0.011 (0.0084 - 0.013) | |
| Pre-enrollment quarter -5 | -39.2 (-54.7 - - 23.7) | | 77.0 (56.1 - 97.9) | | 0.0034 (0.0026 - 0.0042) | | 0.0068 (0.0053 - 0.0084) | |
| Pre-enrollment quarter -4 | -3.53 (-22.6 - 15.5) | | 127.9 (103.2 - 152.7) | | 0.0030 (0.0021 - 0.0040) | | 0.0059 (0.0037 - 0.0080) | |
| Pre-enrollment quarter -3 | 34.1 (21.1 - 47.1) | | 53.7 (35.5 - 71.9) | | 0.0020 (0.0015 - 0.0025) | | 0.0044 (0.0031 - 0.0057) | |
| Pre-enrollment quarter -2 | -13.2 (-30.2 - 3.92) | | 78.5 (56.6 - 100.4) | | 0.0017 (0.00097 - 0.0023) | | 0.0027 (0.00087 - 0.0045) | |
| Post-enrollment quarter 1 | 43.9 (30.9 - 56.9) | | -42.3 (-60.3 - - 24.3) | | -0.0020 (-0.0025 - - 0.0014) | | -0.00075 (-0.0021 - 0.00057) | |
| Post-enrollment quarter 2 | 19.2 (1.02 - 37.4) | 24.7 (0.1-49.2) | -53.0 (-74.8 - - 31.2) | -79.6 (-110.2 - - 50.0) | -0.0039 (-0.0047 - - 0.0032) | -0.006 (-0.008-- 0.004) | -0.0078 (-0.0097 - - 0.0060) | -0.018 (-.020 - - 0.016) |
| Post-enrollment quarter 3 | 91.2 (74.2 - 108.1) | | -96.0 (-117.0 - - 75.1) | | -0.0053 (-0.0060 - - 0.0045) | | -0.0099 (-0.011 - - 0.0083) | |
| Post-enrollment quarter 4 | -69.4 (-89.5 - - 49.4) | -101.9 (-127.2 - - 76.7) | -237.0 (-261.4 - - 212.6) | -244.3 (-276.7 - - 211.9) | -0.0075 (-0.0086 - - 0.0065) | -0.009 -0.011-- 0.007) | -0.019 (-0.022 - - 0.017) | -0.024 (-0.026 - - 0.022) |
| Post-enrollment quarter 5 | -36.3 (-52.4 - - 20.2) | | -299.0 (-320.4 - - 277.5) | | -0.011 (-0.011 - - 0.0096) | | -0.021 (-0.023 - - 0.020) | |
| Post-enrollment quarter 6 | -99.2 (-117.9 - - 80.5) | | -326.7 (-351.8 - - 301.5) | | -0.0077 (-0.0089 - - 0.0065) | | -0.031 (-0.034 - - 0.029) | |
| Post-enrollment quarter 7 | -159.8 (-175.7 - - 143.9) | -328.2 (-355.7 - - 300.7) | -541.2 (-564.2 - - 518.2) | -609.1 (-643.7 - - 574.4) | -0.013 (-0.014 - -0.012) | -0.013 (-0.019-- 0.015) | -0.034 (-0.035 - - 0.032) | -0.026 (-0.028 - - 0.024) |
| Post-enrollment quarter 8 | 241.4 (219.4 - 263.4) | | 102.0 (73.8 - 130.3) | | -0.0068 (-0.0081 - - 0.0054) | | -0.043 (-0.046 - - 0.040) | |
| Post-enrollment quarter 9 | 145.7 (128.1 - 163.2) | | -193.4 (-218.3 - - 168.5) | | -0.012 (-0.013 - -0.011) | | -0.043 (-0.045 - - 0.041) | |

© 2026 Becker NV et al. *JAMA Network Open*.

| | Medical debt in collections | | Total balance on collections | | Total number of public record bankruptcies | | Subprime credit score | |
|---|---|---|---|---|---|---|---|---|
| | Replication result | Miller result | Replication result | Miller result | Replication result | Miller result | Replication result | Miller result |
| Post-enrollment quarter 10 | 551.1 (526.2 - 575.9) | | 495.8 (465.6 - 526.0) | | -0.0062 (-0.0077 - -0.0048) | | -0.052 (-0.055 - -0.049) | |
| Post-enrollment quarter 11 | 709.0 (686.7 - 731.2) | | 466.9 (439.7 - 494.2) | | -0.012 (-0.013 - -0.010) | | -0.054 (-0.056 - -0.052) | |
| Post-enrollment quarter 12 | 491.5 (467.1 - 515.9) | | 459.6 (429.3 - 489.9) | | -0.0061 (-0.0077 - -0.0046) | | -0.061 (-0.064 - -0.059) | |
| Post-enrollment quarter 13 | 654.5 (632.7 - 676.3) | | 431.1 (403.5 - 458.8) | | -0.013 (-0.014 - -0.011) | | -0.064 (-0.066 - -0.062) | |
| Post-enrollment quarter 14 | 397.1 (372.8 - 421.3) | | 468.1 (322.8 - 613.3) | | -0.0070 (-0.0086 - -0.0053) | | -0.071 (-0.074 - -0.068) | |
| Post-enrollment quarter 15 | 526.7 (506.2 - 547.3) | | 321.1 (294.3 - 347.8) | | -0.015 (-0.016 - -0.013) | | -0.071 (-0.073 - -0.069) | |
| Post-enrollment quarter 16 | 310.4 (286.5 - 334.3) | | 318.7 (288.1 - 349.3) | | -0.0080 (-0.0098 - -0.0063) | | -0.080 (-0.083 - -0.078) | |
| Post-enrollment quarter 17 | 388.7 (369.8 - 407.6) | | 202.3 (176.2 - 228.5) | | -0.017 (-0.018 - -0.015) | | -0.082 (-0.084 - -0.080) | |
| Post-enrollment quarter 18 | 216.2 (192.5 - 239.8) | | 207.6 (176.9 - 238.3) | | -0.010 (-0.012 - -0.0082) | | -0.091 (-0.093 - -0.088) | |
| Post-enrollment quarter 19 | 258.2 (240.4 - 276.1) | | 56.6 (30.9 - 82.4) | | -0.019 (-0.021 - -0.018) | | -0.089 (-0.091 - -0.087) | |
| Post-enrollment quarter 20 | 129.8 (106.1 - 153.6) | | 104.8 (73.7 - 136.0) | | -0.013 (-0.015 - -0.011) | | -0.10 (-0.11 - -0.100) | |
| Post-enrollment quarter 21 | 131.0 (112.8 - 149.1) | | -83.6 (-110.1 - -57.1) | | -0.023 (-0.024 - -0.021) | | -0.10 (-0.10 - -0.099) | |
| Post-enrollment quarter 22 | 62.9 (38.3 - 87.5) | | 19.9 (-12.1 - 52.0) | | -0.018 (-0.020 - -0.015) | | -0.13 (-0.13 - -0.13) | |
| Post-enrollment quarter 23 | 30.8 (12.3 - 49.3) | | -198.3 (-225.0 - -171.6) | | -0.028 (-0.030 - -0.026) | | -0.12 (-0.12 - -0.12) | |
| Post-enrollment quarter 24 | -14.1 (-38.7 - 10.4) | | -88.9 (-121.0 - -56.7) | | -0.021 (-0.024 - -0.019) | | -0.17 (-0.17 - -0.16) | |

© 2026 Becker NV et al. *JAMA Network Open*.

| | Medical debt in collections | | Total balance on collections | | Total number of public record bankruptcies | | Subprime credit score | |
|---|---|---|---|---|---|---|---|---|
| | Replication result | Miller result | Replication result | Miller result | Replication result | Miller result | Replication result | Miller result |
| Post-enrollment quarter 25 | -86.4 (-104.2 - -68.5) | | -337.6 (-364.1 - -311.1) | | -0.032 (-0.034 - -0.031) | | -0.16 (-0.16 - -0.16) | |
| Post-enrollment quarter 26 | -73.7 (-96.8 - -50.6) | | -198.5 (-229.6 - -167.4) | | -0.026 (-0.028 - -0.024) | | -0.20 (-0.20 - -0.19) | |
| Post-enrollment quarter 27 | -145.9 (-163.9 - -128.0) | | -444.9 (-471.3 - -418.6) | | -0.037 (-0.039 - -0.035) | | -0.19 (-0.19 - -0.19) | |
| Post-enrollment quarter 28 | -145.4 (-174.8 - -116.0) | | -320.4 (-359.5 - -281.4) | | -0.028 (-0.031 - -0.025) | | -0.22 (-0.22 - -0.21) | |
| Post-enrollment quarter 29 | -211.4 (-230.9 - -192.0) | | -544.1 (-572.7 - -515.4) | | -0.043 (-0.045 - -0.041) | | -0.22 (-0.22 - -0.21) | |
| Observations (N) | 5622153 | | 5622153 | | 5622153 | | 5622153 | |

© 2026 Becker NV et al. *JAMA Network Open*.

**eTable 3.** Regression Coefficients From Primary Analysis

| | Medical debt in collections | Non-medical debt in collections | Any bankruptcy filed in past 2 yr | Subprime credit score |
|---|---|---|---|---|
| Linear pre/post quarter trend | 6.69 (3.36 - 10.0) | -26.8 (-30.4 - -23.1) | -0.00043 (-0.00059 - -0.00028) | -0.0037 (-0.0041 - -0.0033) |
| Linear pre/post quarter trend x 2015 enrollment year | -3.37 (-4.45 - -2.29) | 2.77 (1.61 - 3.93) | 0.000055 (0.0000046 - 0.00011) | -0.00081 (-0.00094 - - 0.00068) |
| Linear pre/post quarter trend x 2016 enrollment year | -5.45 (-7.05 - -3.84) | 4.86 (3.03 - 6.69) | -0.0000014 (-0.000079 - 0.000076) | -0.0015 (-0.0017 - -0.0013) |
| Linear pre/post quarter trend x 2017 enrollment year | -7.77 (-9.88 - -5.65) | 7.77 (5.33 - 10.2) | 0.000042 (-0.000060 - 0.00014) | -0.0019 (-0.0021 - -0.0016) |
| Post-enrollment quarter 1 | -0.92 (-12.3 - 10.4) | 5.99 (-4.68 - 16.7) | 0.00017 (-0.00040 - 0.00073) | 0.0013 (0.000037 - 0.0026) |
| Post-enrollment quarter 2 | 8.79 (-4.44 - 22.0) | 20.7 (8.28 - 33.1) | 0.00073 (0.000036 - 0.0014) | 0.00065 (-0.00092 - 0.0022) |
| Post-enrollment quarter 3 | 32.9 (17.8 - 47.9) | 32.9 (18.5 - 47.2) | 0.00093 (0.00015 - 0.0017) | -0.0020 (-0.0036 - -0.00030) |
| Post-enrollment quarter 4 | -27.9 (-44.5 - -11.3) | 35.3 (18.7 - 51.9) | 0.00087 (-0.000058 - 0.0018) | -0.0097 (-0.012 - -0.0077) |
| Post-enrollment quarter 5 | -61.3 (-80.1 - -42.5) | 25.3 (6.67 - 43.9) | 0.00074 (-0.00029 - 0.0018) | -0.014 (-0.017 - -0.012) |
| Post-enrollment quarter 6 | -64.5 (-85.0 - -44.0) | 55.6 (34.6 - 76.7) | 0.0014 (0.00020 - 0.0025) | -0.022 (-0.025 - -0.020) |
| Post-enrollment quarter 7 | -146.4 (-169.3 - -123.5) | 36.0 (12.5 - 59.5) | 0.00054 (-0.00071 - 0.0018) | -0.026 (-0.029 - -0.024) |
| Post-enrollment quarter 8 | -101.9 (-127.6 - -76.3) | 96.8 (27.5 - 166.2) | 0.0011 (-0.00031 - 0.0025) | -0.038 (-0.041 - -0.035) |
| Post-enrollment quarter 9 | -95.6 (-124.2 - -67.0) | 47.5 (18.2 - 76.9) | 0.00031 (-0.0012 - 0.0018) | -0.039 (-0.042 - -0.036) |
| Post-enrollment quarter 10 | -131.5 (-162.7 - -100.3) | 72.2 (40.3 - 104.1) | 0.00059 (-0.0010 - 0.0022) | -0.050 (-0.054 - -0.047) |
| Post-enrollment quarter 11 | -78.8 (-114.8 - -42.8) | 31.7 (-3.94 - 67.4) | -0.00048 (-0.0022 - 0.0013) | -0.056 (-0.060 - -0.052) |
| Post-enrollment quarter 12 | -173.4 (-210.3 - -136.4) | 68.8 (29.8 - 107.7) | -0.00015 (-0.0020 - 0.0017) | -0.067 (-0.071 - -0.062) |
| Post-enrollment quarter 13 | -136.0 (-178.4 - -93.6) | 47.2 (4.59 - 89.8) | -0.0011 (-0.0032 - 0.00090) | -0.071 (-0.076 - -0.066) |
| Post-enrollment quarter 14 | -234.1 (-277.2 - -191.0) | 100.0 (27.8 - 172.1) | -0.0010 (-0.0032 - 0.0011) | -0.082 (-0.087 - -0.077) |
| Post-enrollment quarter 15 | -197.8 (-247.2 - -148.3) | 22.6 (-30.4 - 75.6) | -0.0022 (-0.0046 - 0.00013) | -0.088 (-0.094 - -0.083) |
| Post-enrollment quarter 16 | -296.9 (-346.5 - -247.3) | 72.2 (20.1 - 124.2) | -0.0016 (-0.0040 - 0.00083) | -0.099 (-0.11 - -0.094) |
| Post-enrollment quarter 17 | -313.9 (-370.0 - -257.8) | 44.2 (-15.3 - 103.8) | -0.0028 (-0.0055 - -0.00012) | -0.10 (-0.11 - -0.097) |
| Post-enrollment quarter 18 | -365.8 (-422.2 - -309.4) | 66.5 (7.55 - 125.4) | -0.0019 (-0.0047 - 0.00085) | -0.12 (-0.12 - -0.11) |

© 2026 Becker NV et al. *JAMA Network Open*.

| | Medical debt in collections | Non-medical debt in collections | Any bankruptcy filed in past 2 yr | Subprime credit score |
|---|---|---|---|---|
| Post-enrollment quarter 19 | -380.4<br>(-445.1 - -315.7) | 36.6<br>(-30.0 - 103.3) | -0.0034<br>(-0.0066 - -0.00034) | -0.12<br>(-0.13 - -0.12) |
| Post-enrollment quarter 20 | -450.6<br>(-514.9 - -386.3) | 69.8<br>(1.80 - 137.7) | -0.0021<br>(-0.0052 - 0.0010) | -0.13<br>(-0.14 - -0.13) |
| Post-enrollment quarter 21 | -516.9<br>(-588.7 - -445.0) | 44.4<br>(-30.4 - 119.3) | -0.0032<br>(-0.0067 - 0.00028) | -0.14<br>(-0.15 - -0.13) |
| Post-enrollment quarter 22 | -525.8<br>(-598.0 - -453.6) | 72.2<br>(-5.40 - 149.9) | -0.0023<br>(-0.0058 - 0.0012) | -0.16<br>(-0.17 - -0.15) |
| Post-enrollment quarter 23 | -610.7<br>(-692.2 - -529.3) | 49.0<br>(-37.6 - 135.7) | -0.0031<br>(-0.0070 - 0.00086) | -0.16<br>(-0.17 - -0.15) |
| Post-enrollment quarter 24 | -638.2<br>(-719.4 - -557.0) | 90.5<br>(2.98 - 178.0) | -0.0024<br>(-0.0064 - 0.0015) | -0.18<br>(-0.19 - -0.17) |
| Post-enrollment quarter 25 | -768.3<br>(-857.5 - -679.2) | 62.4<br>(-32.7 - 157.5) | -0.0036<br>(-0.0079 - 0.00077) | -0.19<br>(-0.20 - -0.18) |
| Post-enrollment quarter 26 | -720.9<br>(-810.7 - -631.0) | 88.0<br>(-11.0 - 187.0) | -0.0027<br>(-0.0071 - 0.0018) | -0.20<br>(-0.21 - -0.19) |
| Post-enrollment quarter 27 | -872.6<br>(-973.5 - -771.7) | 79.1<br>(-30.4 - 188.5) | -0.0031<br>(-0.0080 - 0.0018) | -0.21<br>(-0.22 - -0.20) |
| Post-enrollment quarter 28 | -867.3<br>(-967.7 - -766.9) | 93.8<br>(-18.2 - 205.7) | -0.0031<br>(-0.0079 - 0.0018) | -0.23<br>(-0.24 - -0.22) |
| Post-enrollment quarter 29 | -983.0<br>(-1090.8 - -875.1) | 76.2<br>(-40.6 - 193.0) | -0.0045<br>(-0.0097 - 0.00074) | -0.23<br>(-0.25 - -0.22) |
| Observations (N) | 10119194 | 10119194 | 10119194 | 10119194 |

© 2026 Becker NV et al. *JAMA Network Open*.

**eFigure 9.** Regression Results From “Fully Flexible” Event Study Specification

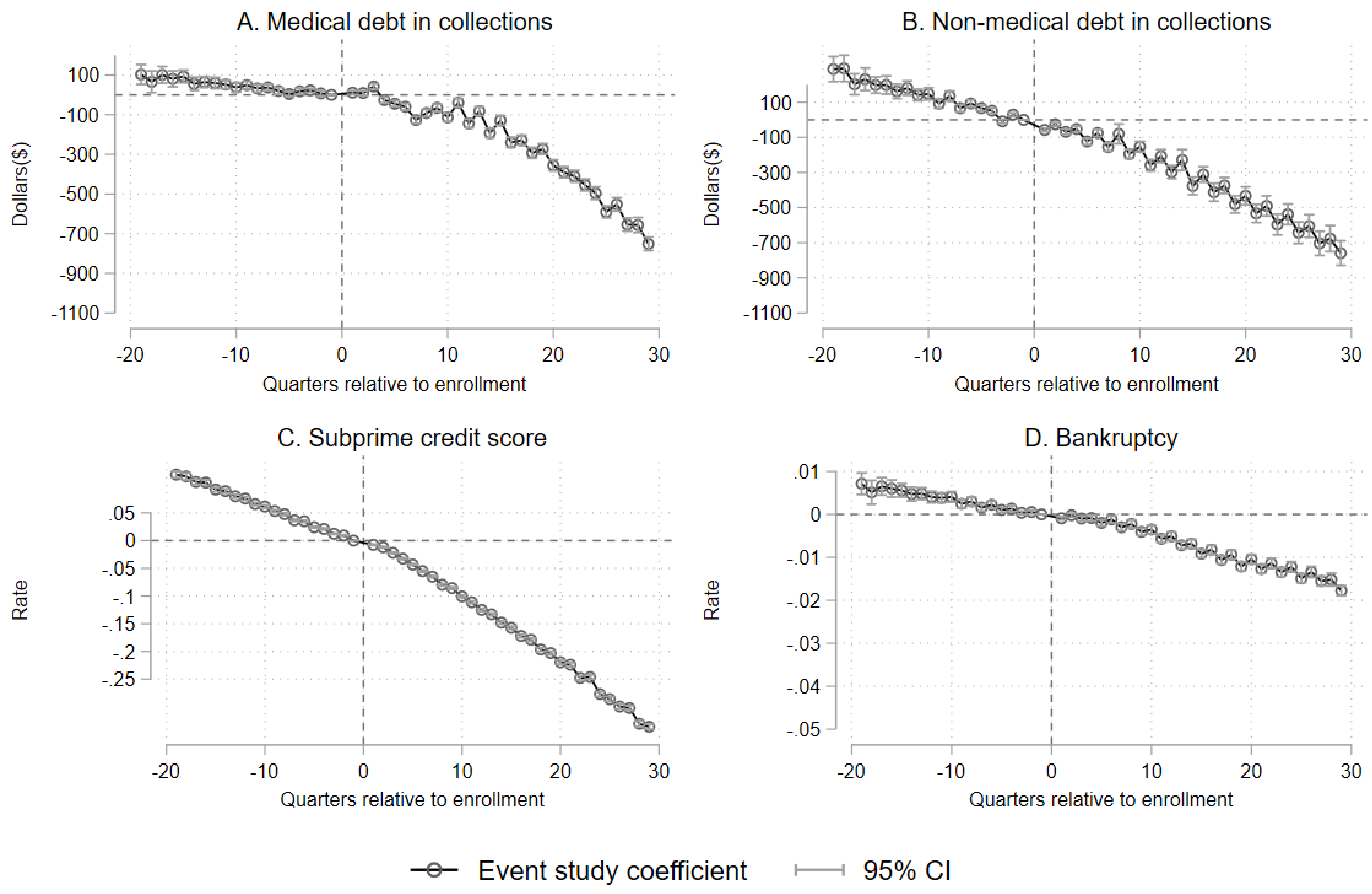


Note: This figure reports results from the model specification described in Equation 2 in the Supplemental Methods.

© 2026 Becker NV et al. *JAMA Network Open*.

**eFigure 10.** Regression Results From Alternative Model Specifications

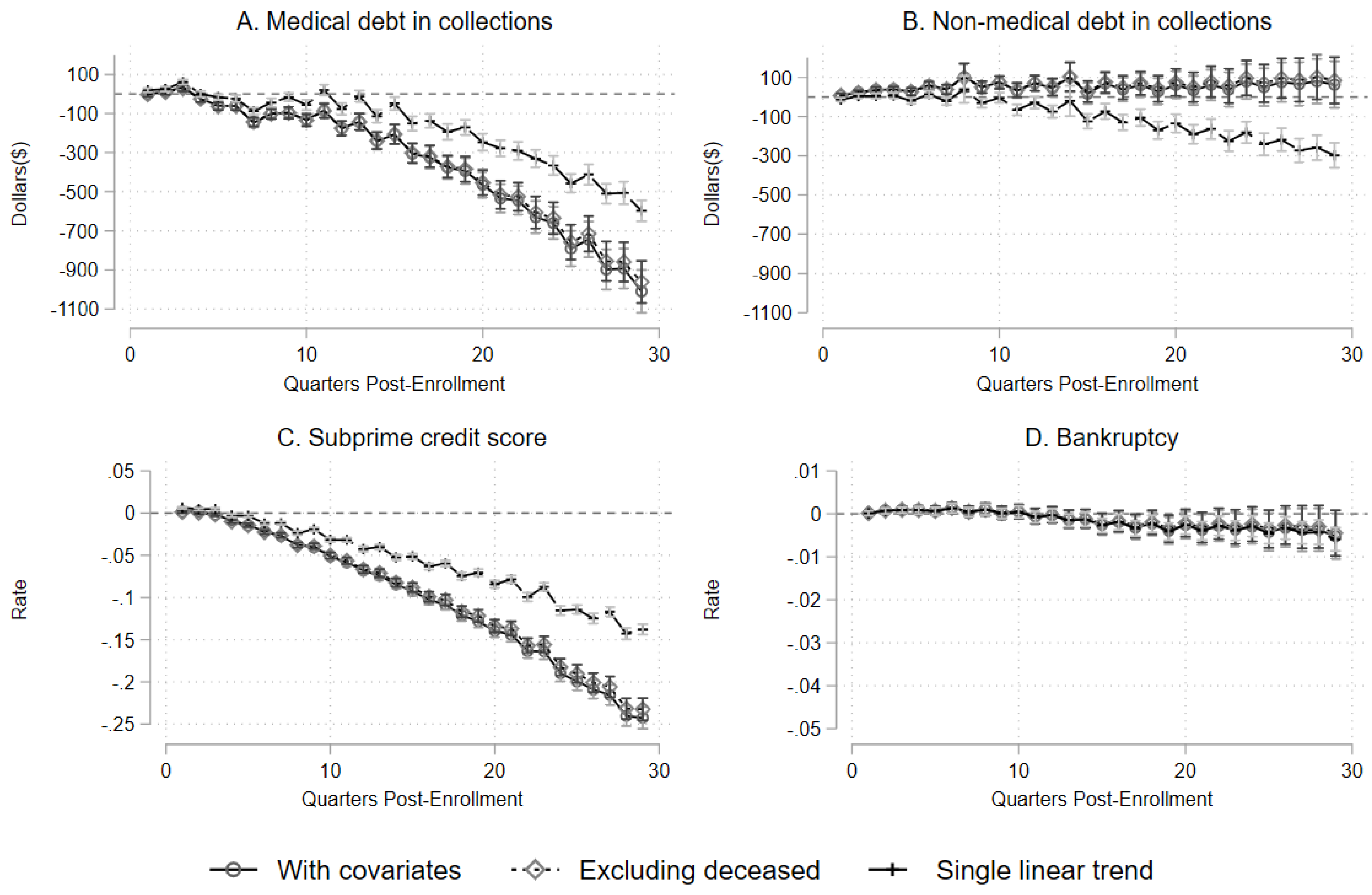


Note: This figure reports results from alternative model specifications described in the Supplemental Methods. The models "with covariates" and "excluding deceased" used the specification outlined in Equation 1; the model "single linear trend" used the specification described in Equation 3.

© 2026 Becker NV et al. *JAMA Network Open*.

**eFigure 11.** Regression Results From 2014-2015 and 2016-2017 Enrollee Subgroups

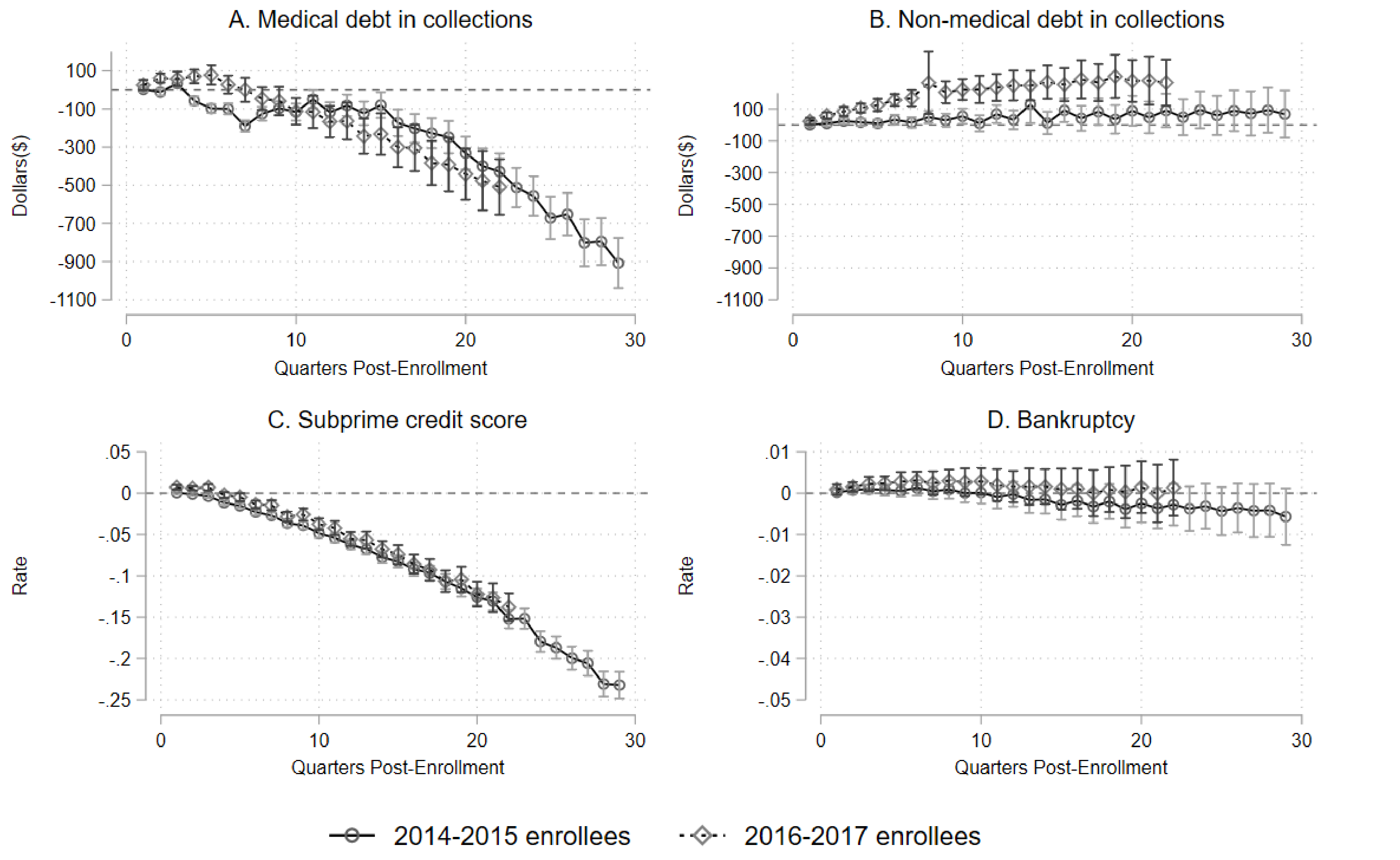


© 2026 Becker NV et al. *JAMA Network Open*.
