# Supplementary material for: Financial Outcomes Among Medicaid Expansion Enrollees
Source: JAMA Netw Open. 2026 Apr 27;9(4):e269328. doi: 10.1001/jamanetworkopen.2026.9328 (PMC13122400; doi:10.1001/jamanetworkopen.2026.9328)
Supplement: Supplement 2. — Data Sharing Statement [file jamanetwopen-e269328-s002.pdf]

Data Sharing Statement

Becker. Financial Outcomes Among Medicaid Expansion Enrollees. *JAMA Netw Open*. Published April 27, 2026. doi:10.1001/jamanetworkopen.2026.9328

Data
Data available: No

Additional Information
Explanation for why data not available: Our data use agreements with the Michigan Department of Health and Human Services and the credit agency do not permit us to share the data used in this study.
